# The Predictive Value of Real‐World Cardiologist Performance (RWCP) Score in Atrial Fibrillation Recurrence Risk After Radiofrequency Ablation

**DOI:** 10.1111/jce.70067

**Published:** 2025-08-19

**Authors:** Yangyang Wang, Jingchao He, Yang Shao, Tong Liu, Aolin Ding, Guohua Xue, Yubing Guo, Pengyu Wu, Yunfei Gu, Hao Wang

**Affiliations:** ^1^ Department of Cardiology Luoyang Central Hospital Affiliated to Zhengzhou University Luoyang Henan Province China; ^2^ Department of Cardiology Sahlgrenska University Hospital Gothenburg Sweden; ^3^ Department of Cardiology, Tianjin Key Laboratory of Ionic‐Molecular Function of Cardiovascular Disease, Tianjin Institute of Cardiology The Second Hospital of Tianjin Medical University Tianjin China; ^4^ Biosense Webster Medical Technology Luoyang Henan Province China

**Keywords:** atrial fibrillation, prediction, radiofrequency catheter ablation, recurrence

## Abstract

**Objective:**

To explore the predictive value of Real‐world Cardiologist Performance (RWCP) score for atrial fibrillation (AF) recurrence risk after radiofrequency catheter ablation (RFCA).

**Methods:**

Patients with nonvalvular AF who underwent first‐time RFCA at Luoyang Central Hospital affiliated to Zhengzhou University between October 2021 and June 2023 were included in this study. Detailed ablation data were exported from CARTO®3 system (Biosense Webster, USA), based on which, four parameters, including discharge time proportion, Catheter Contact Force Stability (C3‐FOT proportion), Ablation Index Consistency (SURPOINT/TOP‐4 proportion) and fragmented points proportion, were calculated. Then, the RWCP score was calculated from these parameters for each patient. Predictive value of the RWCP score for AF recurrence risk were assessed using Cox regression and Kaplan–Meier analyses.

**Results:**

A total of 148 AF patients were enrolled, including 68 males and 80 females. During follow‐up, 30 patients (20.3%) experienced AF recurrence. The RWCP score was calculated using Cox regression coefficients: RWCP score = (left ring discharge time ratio × 2.853) − (left C3‐FOT proportion × 0.91) + (left SURPOINT/TOP4 × 2.943) + (left fragmented points proportion × 0.423) − (right ring discharge time ratio × 4.039) − (right C3‐FOT proportion × 6.159) − (right SURPOINT/TOP4 × 1.312) − (right points fragmented proportion × 4.425). It was found that patients with higher RWCP scores (−12.53 to 5.79) had higher recurrence risk than those with low scores (−28.68 to −12.76) (HR = 5.55, 95% CI: 2.23–13.80, *p* = 0.0002). Kaplan–Meier analysis confirmed this (*p*_log‐rank_ < 0.001). To simplify the operation of RWCP, an online calculator was explored using shiny platform (https://doctorwanghao.shinyapps.io/RWCP_English/).

**Conclusion:**

The RWCP score shows promise as a tool for evaluating ablation completeness, assessing cardiologist performance and predicting AF recurrence risk after ablation.

## 1 Introduction

Atrial fibrillation (AF), the most common arrhythmia in clinical practice [1], increases the risk of adverse clinical events such as ischemic stroke, heart failure, and cognitive impairment by at least two times compared to healthy individuals [2]. It has emerged as a significant global public health concern, seriously threatening human health and life [2, 3]. Currently, catheter ablation is recommended as a first‐line treatment for AF in various guidelines, and circumferential pulmonary vein isolation (CPVI) is recognized as the fundamental ablation strategy [4]. However, the as high as 10%–50% of recurrent rate after ablation significantly limits the long‐term prognostic benefits of ablation for AF patients [5, 6].

In addition to well‐documented patient‐related recurrence risk factors, such as age, alcohol consumption, comorbidities (e.g. hypertension, diabetes mellitus), and atrial fibrosis or low voltage areas [7], numerous studies have attempted to predict the risk of AF recurrence using ablation‐related indicators during CPVI procedures [8, 9, 10, 11], including catheter contact force, ablation index (AI), lesion distance, power and first‐pass isolation (FPI). However, there remains a lack of systematic, comprehensive indicators or multi‐factored evaluation systems to assess the completeness of CPVI procedures [12], which is an essential factor that significantly affects the prognosis of AF after ablation [13, 14, 15, 16, 17]. Even for experienced cardiologists, the completeness of bilateral pulmonary vein isolation rings can inevitably vary. Therefore, the current study aims to develop a score to evaluated CPVI completeness in real‐world conditions.

## 2 Methods

### 2.1 Ethics and Informed Consent

This study complied with the principles of the Declaration of Helsinki and was reviewed and approved by the Medical Ethics Committee of Luoyang Central Hospital Affiliated to Zhengzhou University (ID: LWLL‐2021‐09‐20‐01). All enrolled participants provided written informed consent, who voluntarily agreed to participate in the study.

### 2.2 Study Population

Patients with AF scheduled to undergo first‐time RFCA at our cardiac center between October 2021 and June 2023 were consecutively enrolled. The inclusion criteria were: (i) age > 18 years; (ii) diagnosed as non‐valvular AF according to the 2016 ESC/ECTS Guidelines for the diagnosis and management of atrial fibrillation [2]; (iii) accepting first‐time CPVI treatment; and (iv) providing fully informed consent and voluntary participation. The exclusion criteria were: (i) a history of previous left atrial ablation or cardiac surgery; (ii) equipped with a pacemaker, implantable cardioverter‐defibrillator (ICD), cardiac resynchronization therapy pacemaker (CRT‐P), cardiac resynchronization therapy defibrillator (CRT‐D), or similar devices; (iii) liver dysfunction, defined as total bilirubin or alkaline phosphatase exceeding twice the upper limit of normal, or aspartate aminotransferase (AST) and/or alanine aminotransferase (ALT) exceeding three times the lower limit of normal; and (iv) renal insufficiency, defined as an estimated glomerular filtration rate (eGFR) < 30 mL/min/1.73 m^2^ calculated using the Modified Diet in Renal Disease (MDRD) equation.

### 2.3 Radiofrequency Catheter Ablation (RFCA)

All antiarrhythmic drugs were discontinued for at least five half‐lives previous to ablation. The procedure was performed under fentanyl‐conscious sedation. The CARTO®3 system (Biosense Webster, USA) was utilized for mapping and ablation. Under X‐ray guidance, a 10‐pole mapping catheter was placed in the coronary sinus via the right internal jugular vein. After successful transseptal puncture, an appropriate dose of heparin was administered to maintain an activated clotting time (ACT) of 300–350 s. A Swartz sheath (L1 type, St. Jude Medical, St. Paul, Minnesota, USA) was introduced into the left atrium. Fast anatomical mapping (FAM) of the left atrium and bilateral pulmonary veins was performed using a PentaRay® catheter (Biosense Webster Inc). CPVI was then carried out using Thermocool SMARTTOUCH® SF catheter, with a power output of 45 W, a cold heparinized saline flushing rate of 15 mL/min, and a contact force maintained within 10 ± 5 g. The target ablation index (AI) value was 480 ± 10 for the anterior wall of the bilateral pulmonary veins, 450 ± 10 for the roof and bottom, 400 ± 10 and 380 ± 10 for the posterior wall of the right and left pulmonary veins correspondingly. The procedural endpoint was defined as bidirectional electrical isolation of CPVI rings, confirmed after a 30‐min observation.

### 2.4 Ablation‐Related Parameters Definitions and the RWCP Score Calculation

Detailed ablation‐related data were extracted from CARTO®3 system (Biosense Webster, USA) and transformed into the following parameters: discharge time proportion, C3‐FOT proportion, SURPOINT/TOP‐4 proportion and fragmented points proportion, which were first proposed and defined by Professor Long Chen [18]. Among them, discharge time proportion is defined as the total RFCA discharge time during CPVI divided by the total CPVI procedure time, indicating the difficulty of catheter positioning during procedure; Catheter Contact Force Stability (C3‐FOT proportion) refers to the percentage of time during the ablation procedure at a given point where the contact force maintains above 4 g for 30% of every 2.5‐s interval, reflecting the stability of contact force during ablation; Ablation Index Consistency (SURPOINT/TOP‐4 proportion) represents the percentage of ablation points on the ipsilateral ablation ring with AI values concentrated within the top four AI value ranges, indicating the level of dispersion of AI values during the ablation process; while, fragmented points proportion is calculation as the ratio of fragmented points generated by significant catheter movement, which is defined as a movement exceeding 3 mm and lasting for more than 3 s, which reflects catheter stability during ablation. The parameters for both the left and right CPVI rings were collected after ablation and then assigned score from 1 to 3 points based on their corresponding third percentiles. Subsequently, these indicators were transformed into ordered categorical variables and incorporated into a Cox regression model to derive the regression coefficients for each parameter.

It is widely accepted [19] that patients with paroxysmal atrial fibrillation (PAF) primarily caused by trigger mechanisms, which CPVI predominantly addresses, while patients with persistent atrial fibrillation (PsAF) may primarily have maintenance mechanisms driven by atrial substrate fibrosis. In addition to defining patient categorization based on whether the duration of atrial fibrillation exceeds 7 days (the clinical criteria for paroxysmal and persistent AF), we also employed high‐density low‐voltage mapping results obtained from the PentaRay catheter. Patients were classified based on previously reported [20] standards of substrate fibrosis: the low‐voltage standard (local bipolar voltage < 0.5 mV under sinus rhythm mapping) and significant fibrosis standard (left atrial low‐voltage area > 20%). Therefore, we also conducted separate analyses in patients with PAF and those without significant fibrosis (left atrial low‐voltage area percentage < 20%).

### 2.5 Follow‐Up

Regular follow‐up was performed via outpatient visits at 30 ± 7, 90 ± 7, 180 ± 7, and 360 ± 7 days after discharge, followed by subsequent evaluations every 180 ± 7 days. Hematological tests, electrocardiograms (ECG) and holter electrocardiography were scheduled at each visit. Echocardiography was conducted at every 6‐month visit. For patients with suspicious arrhythmia symptoms, 24–72‐h dynamic electrocardiogram monitoring or 30‐day event monitoring was performed based on symptom frequency. According to the 2012 HRS/EHRA/ECAS Expert Consensus Statement on Catheter and Surgical Ablation of Atrial Fibrillation, AF recurrence was defined as any symptomatic or asymptomatic atrial tachyarrhythmia (AF, atrial flutter, or atrial tachycardia) lasting more than 30 s or detected on a 12‐lead ECG (10 s), after a 3‐month blanking period [21].

### 2.6 Statistical Analysis

Continuous variables were presented as mean ± standard deviation (SD), while categorical variables were expressed as frequency (percentage). Depending on whether the data follow a normal distribution, the independent two‐sample *t*‐test or Mann–Whitney *U* test was applied. For categorical variables, the chi‐square test or Fisher's exact test was used according to the presence of expected counts less than five. Univariate and multivariate Cox regression was utilized to identify clinical factors associated with AF recurrence. The relative risk was expressed as hazard ratios (HR) with 95% confidence intervals (CI). Variables included in the multivariate Cox regression model were selected based on the following criteria: (i) factors previously reported in previous literatures to be closely associated with AF recurrence, such as sex, age, type of atrial fibrillation onset, and anterior‐posterior diameter of left atrium; (ii) variables that, when added to the model, resulted in more than 10% change in the initial effect estimate (HR value) or had a *p*‐value < 0.1 in the univariate Cox regression analysis. The recurrence‐free rate was evaluated using the Kaplan–Meier method. Statistical analyses were performed using SPSS 23.0 for Windows (Chicago, IL, USA) and R version 4.3.2 (R Foundation for Statistical Computing, Vienna, Austria), with statistical significance set at *p* < 0.05 (two‐sided).

## 3 Results

### 3.1 Baseline Characteristics of Participants

**Figure 1:**
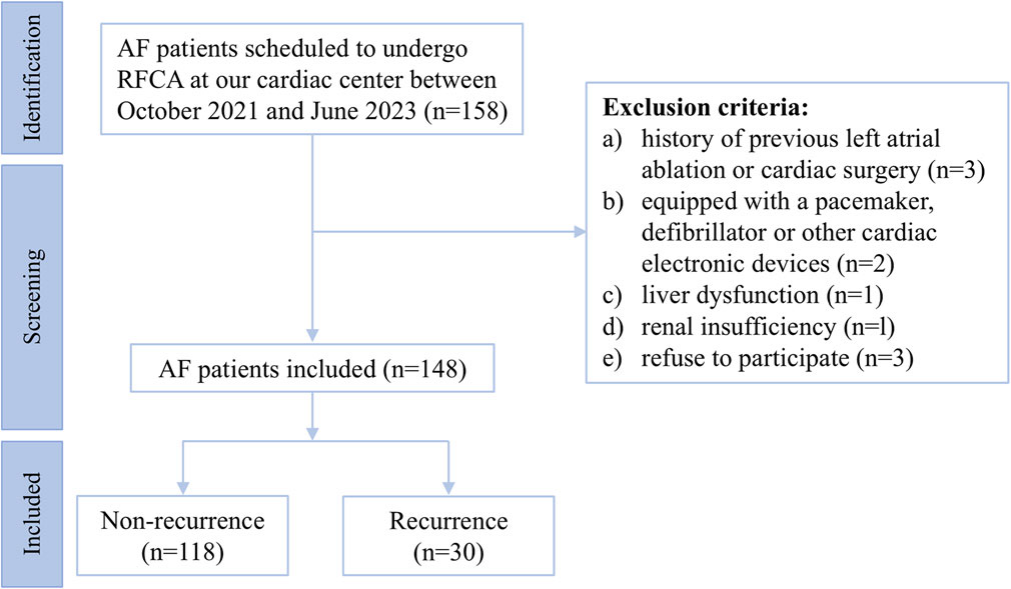
The flow chart of patient enrollment. AF, atrial fibrillation; RFCA, radiofrequency catheter ablation.

The flowchart of patient enrollment is shown in Figure 1. A total of 148 individuals were finally included in this study, with 30 patients (20.3%) experiencing AF recurrence. The average age of the patients was 61.92 ± 2.23 years, and 68 were females (45.9%). A comparison of baseline clinical characteristics and procedure parameters between the recurrence and non‐recurrence groups is detailed in Table 1. As can be seen, patients in the recurrence group were found to be relatively older (*p* = 0.048) and had a significantly lower.

**Table 1:** Baseline information and ablation parameters of patients.

	Nonrecurrence (*n* = 118)	Recurrence (*n* = 30)	*p*‐value
Gender (*n*, %)			0.929
Female	54 (45.76%)	14 (46.67%)	
Male	64 (54.24%)	16 (53.33%)	
Age (year)	62.15 (59.02–64.10)	61.00 (60.00–62.00)	**0.048**
BMI (kg/m^2^)	25.22 ± 3.11	25.73 ± 4.57	0.469
Smoking (*n*, %)	33 (27.97%)	8 (26.67%)	0.887
Drinking (*n*, %)	36 (30.51%)	12 (40.00%)	0.321
HBP (*n*, %)	55 (46.61%)	15 (50.00%)	0.740
DM (*n*, %)	17 (14.41%)	3 (10.00%)	0.528
CAD (*n*, %)	16 (13.56%)	1 (3.33%)	0.117
Stroke/TIA (*n*, %)	6 (5.08%)	2 (6.67%)	0.664
AF onset type (*n*, %)			0.142
PAF	100 (84.75%)	22 (73.33%)	
PsAF	18 (15.25%)	8 (26.67%)	
AF duration (month)	12.00 (2.00–24.00)	12.00 (4.50–30.25)	0.492
TEE parameters			
LAD (mm)	39.34 ± 5.05	38.77 ± 5.13	0.581
LVEDD (mm)	46.21 ± 4.19	45.43 ± 4.09	0.363
LVEF (%)	65.59 ± 5.99	66.37 ± 5.73	0.525
Ablation parameters			
Left ring			
Discharge time proportion	36.90 (29.00–46.13)	39.05 (30.97–45.57)	0.694
C3‐FOT proportion	95.58 ± 10.13	95.22 ± 6.14	0.853
SURPOINT/TOP‐4 proportion	88.02 ± 11.19	89.38 ± 8.05	0.534
Fragmented points proportion	9.36 ± 12.97	6.90 ± 7.80	0.321
Right ring			
Discharge time proportion	36.35 (29.40–46.05)	35.35 (29.05–42.18)	0.441
C3‐FOT proportion	95.46 ± 5.39	92.17 ± 8.74	**0.010**
SURPOINT/TOP‐4 proportion	94.06 ± 7.82	94.71 ± 5.30	0.670
Fragmented points proportion	4.78 ± 9.45	3.74 ± 7.18	0.573
Groups (single‐circle isolation)			0.096
A	25 (21.19%)	3 (10.00%)	
B	21 (17.80%)	9 (30.00%)	
C	24 (20.34%)	10 (33.33%)	
D	48 (40.68%)	8 (26.67%)	

*Note:* Group A, neither side attained single‐circle isolation; Group B, right side did not attain single‐circle isolation while the left did; Group C, left side did not attain single‐circle isolation while the right did; Group D, both sides attained single‐circle isolation. Bold values indicate statistically significant differences (*p* < 0.05).

Abbreviations: AF, atrial fibrillation; BMI, body mass index; CAD, coronary heart disease; DM, diabetes mellitus; HBP, high blood pressure; LAD, left atrial diameter; LVEDD, left ventricular end‐diastolic diameter; LVEF, left ventricular ejection fraction; PAF, paroxysmal atrial fibrillation; PsAF, persistent atrial fibrillation; TIA, transient ischemic attack; TTE, transthoracic echocardiography.

Catheter contact force stability (C3‐FOT proportion) of right ring (*p* = 0.01). Although the fragmented points proportion of right ring in the recurrence group showed a lower trend, the difference did not reach statistical significance. Other indicators showed no significant difference between the two groups (*p* > 0.05).

While the correlation between single‐circle isolation and the risk of atrial fibrillation recurrence remains contentious, many studies have indeed reported a relationship between the two. Based on whether the left or right isolation achieved single‐circle isolation, we classified the patients into four groups: Group A, neither side attained single‐circle isolation; Group B, right side did not attain single‐circle isolation while the left did; Group C, left side did not attain single‐circle isolation while the right did; and Group D, both sides attained single‐circle isolation. There was no significant difference in the achievement of single‐circle isolation between the recurrence and non‐recurrence groups (*p* > 0.05).

### 3.2 The RWCP Score Calculation

Based on the methodology described above, the RWCP score was constructed. Table 2 presents the tertiles and regression coefficients for the parameters of bilateral CPVI rings. Due to the regression coefficient being too small, they were amplified by 10 times to derive the RWCP score formula. The final RWCP score was as follows: RWCP score = (left ring discharge time ratio × 2.853) − (left C3‐FOT proportion × 0.91) + (left SURPOINT/TOP4 × 2.943) + (left fragmented points proportion × 0.423) − (right ring discharge time ratio × 4.039) − (right C3‐FOT proportion × 6.159) − (right SURPOINT/TOP4 × 1.312) − (right fragmented points proportion × 4.425). All radiofrequency ablation procedures were performed by two independent and experienced operators (both with over 10 years of clinical experience). The operators' average RWCP scores were −11.34 ± 4.42 and −11.08 ± 3.89, respectively, with no statistically significant difference between them (*p* = 0.295). Besides, we also compared the RWCP scores across Groups A, B, C, and D, which were stratified based on whether left or right isolation achieved single‐circle isolation. The corresponding results could be seen in Supporting Information: Table S1, which presents the distribution of RWCP scores for each group along with one‐way analysis of variance results, showing no statistically significant differences among the four groups (*p* > 0.05), while, Supporting Information: Table S2 provides detailed intergroup comparisons, confirming no significant differences in RWCP scores between any two groups. We incorporated this factor into multivariate Cox regression analysis and found that the results remained robust after adjustment, as demonstrated in Supporting Information: Table S3.

**Table 2:** The tertiles and regression coefficients for ablation parameters.

Ablation parameters	T1 (*n*, range)	T2 (*n*, range)	T3 (*n*, range)	RC*10
Left ring				
Discharge time proportion	48 (13.90–31.70)	50 (31.80–42.50)	50 (42.60–100.00)	2.853
C3‐FOT proportion	49 (0.00–95.70)	39 (95.80–98.10)	60 (100.00–100.00)	−0.910
SURPOINT/TOP‐4 proportion	49 (14.50–86.40)	49 (86.80–93.50)	50 (93.90–100.00)	2.943
Fragmented points proportion	73 (0.00–2.90)	75 (3.10–85.50)		0.423
Right ring				
Discharge time proportion	49 (19.00–31.80)	49 (31.90–42.20)	50 (42.30–85.50)	−4.039
C3‐FOT proportion	49 (63.50–93.60)	48 (93.70–98.10)	51 (100.00–100.00)	−6.159
SURPOINT/TOP‐4 proportion	49 (35.40–93.40)	48 (93.50–97.40)	51 (97.50–100.00)	−1.312
Fragmented points proportion	96 (0.00–2.20)	52 (2.40–74.40)		−4.425

Abbreviations: C3‐FOT, Catheter Contact Force Stability; RC, regression coefficient; SURPOINT/TOP‐4, Ablation Index Consistency.

### 3.3 Univariate and Multivariate Cox Regression Analyses

Univariate Cox regression analysis did not identify any clinical indicators with statistically significant predictive value for AF recurrence risk (Table 3). Based on well‐established AF recurrence risk factors reported in previous research works, variables such as sex, age, AF onset type, and left atrial diameter (LAD) were included in a multivariable Cox regression for adjustment. The results across all three levels of adjustment, including unadjusted model, minimally adjusted model (adjusting only for sociodemographic factors such as sex and age), and fully adjusted model (adjusting AF onset type and LAD, besides sex and age) were summarized in Table 4. All three adjustment models suggested that RWCP score was an independent predictor of AF recurrence risk. In the fully adjusted model, each one‐point increase in RWCP score was associated with an approximately 10% increase in AF recurrence risk (*p* = 0.0009).

**Table 3:** The results of univariate cox regression analysis.

Parameters	HR (95%CI)	*p*‐value
Male	1.02 (0.50–2.09)	0.958
Age	0.96 (0.90–1.02)	0.207
BMI	1.04 (0.94–1.15)	0.462
Smoking	0.91 (0.40–2.03)	0.810
Drinking	1.20 (0.58–2.50)	0.619
PsAF	1.74 (0.77–3.91)	0.179
AF duration	1.00 (0.99–1.01)	0.434
HBP	1.20 (0.59–2.47)	0.611
DM	1.00 (0.30–3.30)	0.998
CAD	0.29 (0.04–2.13)	0.224
Stroke/TIA	2.52 (0.59–10.69)	0.210
LAD	0.97 (0.90–1.04)	0.431
LVEDD	0.96 (0.88–1.04)	0.314
LVEF	1.04 (0.97–1.11)	0.235
Groups (single‐circle isolation)		
A	1	
B	3.18 (0.86–11.76)	0.083
C	3.62 (0.99–13.16)	0.051
D	1.47 (0.39–5.55)	0.569

*Note:* Group A, neither side attained single‐circle isolation; Group B, right side did not attain single‐circle isolation while the left did; Group C, left side did not attain single‐circle isolation while the right did; Group D, both sides attained single‐circle isolation.

Abbreviations: AF, atrial fibrillation; BMI, body mass index; CAD, coronary heart disease; DM, diabetes mellitus; HBP, high blood pressure; LAD, left atrial diameter; LVEDD, left ventricular end‐diastolic diameter; LVEF, left ventricular ejection fraction; PsAF, persistent atrial fibrillation; TIA, transient ischemic attack.

**Table 4:** Predictive value of RWCP on AF recurrence risk in different models.

Variable	Crude model (HR, 95%CI, *p*‐value)	Minimally adjusted model (HR, 95%CI, *p*‐value)	Fully adjusted model (HR, 95%CI, *p*‐value)
RWCP score (consistent)	1.10 (1.04–1.17) 0.0007	1.11 (1.04–1.17) 0.0005	1.10 (1.04–1.17) 0.0009
RWCP score (tertiles)			
Low (−28.68 to −16.68)	1.0	1.0	1.0
Medium (−16.51 to −9.34)	1.87 (0.68–5.14) 0.2270	2.01 (0.72–5.59) 0.1823	2.19 (0.77–6.22) 0.1420
High (−9.02 to 5.79)	3.05 (1.17–7.95) 0.0227	3.22 (1.23–8.45) 0.0175	3.22 (1.19–8.70) 0.0209
RWCP score (median)			
Low (−28.68 to −12.76)	1.0	1.0	1.0
High (−12.53 to 5.79)	5.35 (2.18–13.12) 0.0002	5.47 (2.23–13.45) 0.0002	5.55 (2.23–13.80) 0.0002

*Note:* Crude model: no covariate adjusted. Minimally adjusted model: only age and sex adjusted. Fully adjusted model: In addition to age and sex, AF onset type and LAD were also adjusted.)

Abbreviations: CI, confidence interval; HR, hazard ratio; LAD, left atrial diameter; RWCP, real‐world cardiologist performance.

**Figure 2:**
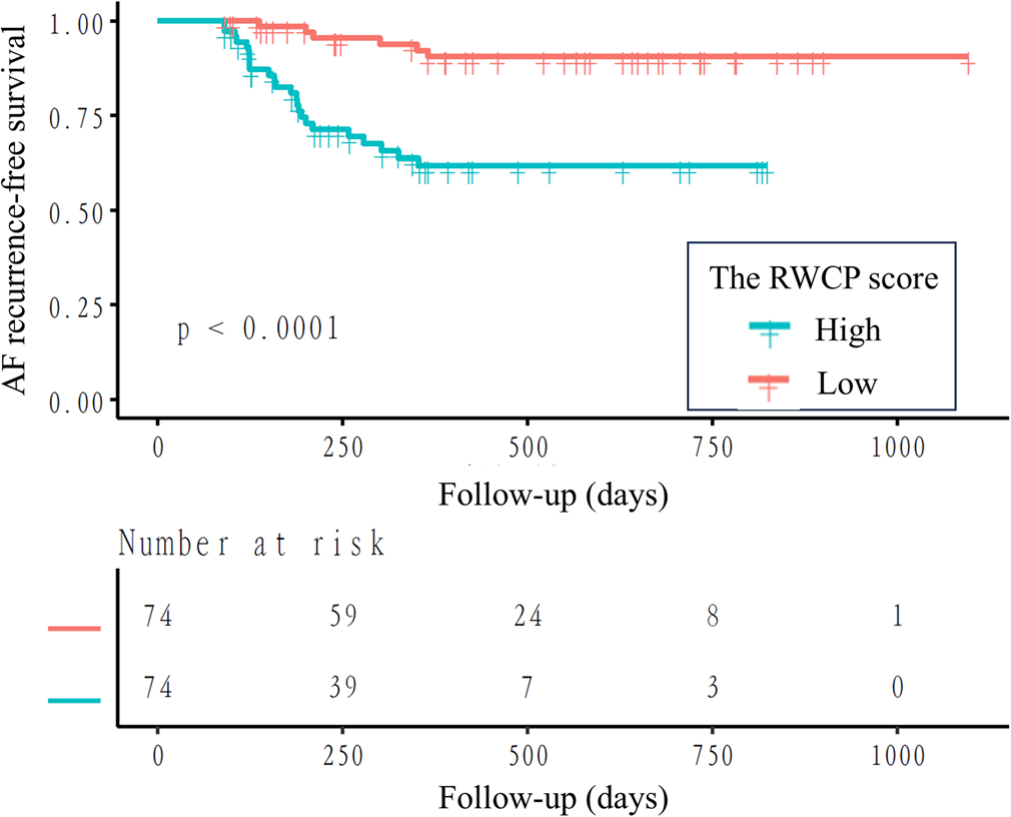
Kaplan–Meier analysis. Patients in the high RWCP score group showed significantly higher recurrence risk than those in the low score group (*p*_log‐rank_ < 0.0001). RWCP, real‐world cardiologist performance.

To facilitate clinical application, patients were further classified into three groups (high, medium and low RWCP score) based on tertiles, as well as two groups (high and low RWCP score) according to median. Kaplan–Meier survival analysis demonstrated a clear trend in survival curves across the three groups (high, medium, and low RWCP score). However, the log‐rank test for the three groups did not reach statistical significance (Supporting Information: Figure 1S). In the two‐group comparison, patients in the high RWCP score group showed significantly higher recurrence risk than those in the low score group, as shown in Figure 2 (*p*_log‐rank_ < 0.0001). To further improve clinical utility, an online RWCP score calculator was developed and released (https://doctorwanghao.shinyapps.io/RWCP_English/). Consistent statistical methods were applied to analyze both PAF patients and those with low‐voltage areas less than 20%. Significant differences (*p* < 0.05) were also observed in all analyses: for PAF patients (Supporting Information: Tables S4–S7 and Figures S2–S3) and for patients with limited low‐voltage areas (Supporting Information: Tables S8–S11 and Figures S4–S5). These results demonstrate that RWCP scores maintain robust predictive value in both PAF patients and those without severe atrial fibrosis.

## 4 Discussion

The primary finding of this study is that RWCP score is an independent predictor for postablation AF recurrence. After adjusting for sex, age, AF onset type and LAD, each one‐point increase in RWCP score was associated with an approximately 10% increase in AF recurrence risk (*p* = 0.0009). And RWCP scores maintain robust predictive value in both PAF patients and those without severe atrial fibrosis. To the best of our knowledge, this appears to be the first report to propose a score that integrates multiple ablation‐related indicators, including catheter accessibility, catheter contact force stability, and AI dispersion, during the ablation procedure.

The RWCP score, proposed here, is calculated based on the discharge time proportion, the Catheter Contact Force Stability (C3‐FOT proportion), the Ablation Index Consistency (SURPOINT/TOP‐4 proportion), and the fragmented points proportion. Specifically, as previously reported, the discharge time proportion was associated with the recurrence risk of AF after ablation, as it indicates the difficulty of catheter positioning to the target site, which could be influenced by the anatomical morphology of the atrium and pulmonary veins [22], patients' breathing amplitude and level of cooperation, as well as the ideal degree of the transseptal puncture site. The C3‐FOT proportion reflects the stability of contact force during the local discharge process at each ablation point, which is closely related to the effective transmission of ablation energy by the catheter [23, 24, 25]. The fragmented points proportion is similar but not identical with C3‐FOT proportion. It indicates the stability of the catheter's position during the discharge process at each ablation point, which directly affects the amount of effective energy received locally at that ablation site. These two indicators are closely related to the effectiveness of ablation. Currently, cool saline‐irrigated catheters with power‐controlled mode are widely used for AF ablation, with a significant correlation between lesion size and tissue contact. In vitro, it has shown that [26], due to the absence of temperature limitation, lesions generated by a 30 g contact force are significantly larger than those by a 10 g contact force (under perpendicular contact and 0.1 m/s saline irrigation, the lesion depth and volume of 30 g were 4.4 ± 0.6 mm and 99 ± 43 mm³, whereas for 10 g, the lesion depth and volume were only 3.0 ± 1.0 mm and 40 ± 33 mm³). In the beating heart, catheter contact force and position are dynamic, instead of constant and unchanging. Shah et al. [27] simulated such dynamic contact using bovine skeletal muscle in an in vitro experiment. It was found that constant (static) contact produced the largest lesions, while unstable and intermittent contact significantly reduced ablation damage. Currently, to improve the stability of catheter positioning and contact force, researchers have attempted to enhance ablation effectiveness through maintaining general anesthesia and applicating steerable long sheaths.

The significantly higher weighting of right‐sided Catheter Contact Force Stability (C3‐FOT proportion) in the RWCP score (coefficient: −6.159 vs. −0.91 for left side) may reflect the anatomical complexity of right pulmonary vein (RPV) ablation. As demonstrated by Ruiz‐Granell et al. [28], right PVs were reconnected more frequently in pre‐RF patients. This anatomical complexity likely increases the technical difficulty of maintaining stable catheter contact during RPV ablation, making contact force stability (as measured by C3‐FOT) particularly crucial on this side. And Nagy et al. [29] and Marom's et al. [30] research found that patients with the RPV anatomical parameters or a separate ostia for the right middle lobe pulmonary vein(s) tended to have a higher frequency of atrial arrhythmia than those with other patterns (*p* = 0.053). Our findings support prioritizing contact force monitoring during right‐sided ablation, consistent with recent clinical reports suggesting that right PV reconnection occurs more frequently than left PV reconnection.

The SURPOINT/TOP‐4 proportion, while, refers to the percentage of ablation points within the same ablation ring where AI values are concentrated in the top four AI ranges. It reflects the concentration and dispersion of AI values. AI (ablation index) is an intraoperative real‐time feedback metric for the effectiveness of ablation, calculated using a weighted formula that incorporates contact force, time, and power [31]. The thickness of the atrial walls varies notebly. For example, the anterior wall and roof of the left atrium is significantly thicker than the posterior and inferior walls [32], thus requiring higher AI values to achieve transmural injury and prevent reconnection. Conversely, certain regions of the atrium require relatively lower AI values due to being close to critical organs, such as the posterior wall of the left superior pulmonary vein, which is adjacent to the esophagus [33, 34]. Considering these factors, the currently recommended AI values are mainly concentrated in four specific ranges: 480 ± 10 for the anterior margins of both pulmonary veins, 450 ± 10 for the roof and bottom, 400 ± 10 for the posterior wall of the right pulmonary vein, and 380 ± 10 for the posterior wall of the left pulmonary vein. Therefore, SURPOINT/TOP‐4 proportion indicates the stability of ablation damage.

Previous studies [35, 36, 37] have demonstrated that first‐pass isolation may influence atrial fibrillation recurrence rates through postablation pulmonary vein reconnection. However, the achievement of FPI is multifactorial, involving both patient‐specific characteristics and procedural variables. Beyond anatomical factors (e.g., variations in pulmonary vein morphology), the presence of left atrial low‐voltage areas may compromise transmural lesion formation, while proximity to critical structures (e.g., esophagus and phrenic nerve) often necessitates reduced ablation energy or duration, potentially affecting lesion continuity. Furthermore, procedural elements such as ablation parameters (power, duration) and catheter stability play pivotal roles.

Compared to established AF recurrence prediction scores, the RWCP score offers unique advantages by incorporating real‐time procedural metrics. The widely used APPLE score (age, persistent AF, impaired eGFR, LA diameter, and EF < 50%) relies solely on preprocedural clinical factors, can evaluate the recurrence of atrial fibrillation after the first PVI operation, and help to screen patients more suitable for atrial fibrillation ablation before operation [38]. Similarly, CAAP‐AF score [39] [coronary atherosclerotic heart disease (CAD, 1 point), left atrial diameter (1–4 points), age (1–3 points), persistent atrial fibrillation (2 points), ineffective antiarrhythmic drugs (1–2 points), female (1 point)] and ATLAS score [40] [age (> 60 years, 1 point), type of atrial fibrillation (2 points), left atrial volume index (1 point for every 10 ml/m^2^ increase), female (4 points) and smoking (7 points)] demonstrates modest predictive value but excludes procedural variables. Moreover, some scholars [41] have found that the predictive value of APPLE and CAAP‐AF score are limited.

The RWCP score represents a paradigm shift from traditional clinical scores by systematically evaluating four key procedural parameters that directly reflect ablation quality. These parameters, comprehensively assess catheter accessibility, contact force stability, ablation index consistency, and catheter positional stability during CPVI. The RWCP formula weights these parameters based on their Cox regression coefficients, creating a standardized tool that objectively quantifies procedural quality.

### 4.1 Limitations

First, the RWCP score is derived from radiofrequency ablation parameters and, therefore, is not applicable for recurrence risk prediction in AF patients accepting alternative energy ablation, such as cryoballoon ablation and pulsed‐field ablation. Second, as patients included in this study all underwent point‐by‐point discharge ablation for CPVI, the applicability of the RWCP score in patients treated with high‐power and continuous discharge ablation techniques requires further validation. Additionally, the single‐center design may introduce potential biases, such as consistency in operator experience and procedural techniques, and the lack of external validation limits the generalizability of our findings. Future multi‐center studies and external validation cohorts are needed to confirm the robustness of our results. Moreover, a common limitation faced by most studies investigating the predictive value of ablation‐related parameters for AF recurrence is the difficulty in obtaining intracardiac mapping results during the second ablation in patients with recurrence, precluding definitive determination of whether recurrence is related to the quality of CPVI ablation. Future studies incorporating patients undergoing second procedures are warranted to provide more direct evidence and further validate our findings. Nevertheless, since CPVI remains the foundational ablation strategy for AF ablation, the value of RWCP score in assessing CPVI quality continues to be substantial.

## 5 Conclusions

The RWCP score shows promise as a tool for evaluating ablation completeness, assessing cardiologist performance and predicting AF recurrence risk after ablation.

## Author Contributions

Yangyang Wang participated in the research design, data collection, data analysis, and writing of the first draft of the paper. Yunfei Gu is responsible for overseeing and coordinating internal communication within the team, as well as handling all external contacts related to research. Hao Wang provided professional guidance in data analysis and paper writing, participated in research design discussions, and provided valuable scientific insights. Aolin Ding is mainly responsible for data collection, while Yang Shao and Tong Liu are mainly responsible for professional guidance. Guohua Xue, Yubing Guo, and Pengyu Wu cover data collection, experimental operations, and statistical analysis.

## Ethics Statement

The research was approved by the Institutional Review Board of Luoyang Central Hospital Affiliated to Zhengzhou University (ID: LWLL‐2021‐09‐20‐01). The informed consents had been obtained from the patients.

## Consent

The informed consents had been obtained from the patients.

## Conflicts of Interest

The authors declare no conflicts of interest.

## Supporting information


**Table S1:** The distribution of RWCP scores for each group along with the results of one‐way ANOVA. **Table S2:** The further inter‐group comparison results. **Table S3:** Included whether the left or right isolation achieved single‐circle isolation in the multivariate Cox regression analysis. **Table S4:** Baseline information and ablation parameters of PAF patients. **Table S5:** The tertiles and regression coefficients for ablation parameters in PAF patients. **Table S6:** The results of univariate cox regression analysis in PAF patients. **Table S7:** PAF patients' predictive value of RWCP on AF recurrence risk. **Table S8:** Baseline information and ablation parameters among patients with low voltage areas less than 20%. **Table S9:** The tertiles and regression coefficients for ablation parameters among patients with low voltage areas less than 20%. **Table S10:** The results of univariate cox regression analysis among patients with low voltage areas less than 20%. **Table S11:** Among patients'with low voltage areas less than 20% predictive value of RWCP on AF recurrence risk in different models.

**Figure S1:** Kaplan‐Meier analysis (grouped by tertiles).

**Figure S2:** The Kaplan‐Meier analysis of two groups in PAF.

**Figure S3:** The Kaplan‐Meier analysis of three groups in PAF.

**Figure S4:** The Kaplan‐Meier analysis of two groups among patients with low voltage areas less than 20%.

**Figure S5:** The Kaplan‐Meier analysis of three groups among patients with low voltage areas less than 20%.

## Data Availability

The data that support the findings of this study are available from the corresponding author upon reasonable request.
