# Supplementary material for: The Predictive Value of Real‐World Cardiologist Performance (RWCP) Score in Atrial Fibrillation Recurrence Risk After Radiofrequency Ablation
Source: J Cardiovasc Electrophysiol. 2025 Aug 19;36(11):2885–93. doi: 10.1111/jce.70067 (PMC12614126; doi:10.1111/jce.70067)
Supplement: Supplementary file 1 — Table S1: The distribution of RWCP scores for each group along with the results of one‐way ANOVA. Table S2: The further inter‐group comparison results. Table S3: Included whether the left or right isolation achieved single‐circle isolation in the multivariate Cox regression analysis. Table S4: Baseline information and ablation parameters of PAF patients. Table S5: The tertiles and regression coefficients for ablation parameters in PAF patients. Table S6: The results of univariate cox regression analysis in PAF patients. Table S7: PAF patients' predictive value of RWCP on AF recurrence risk. Table S8: Baseline information and ablation parameters among patients with low voltage areas less than 20%. Table S9: The tertiles and regression coefficients for ablation parameters among patients with low voltage areas less than 20%. Table S10: The results of univariate cox regression analysis among patients with low voltage areas less than 20%. Table S11: Among patients'with low voltage areas less than 20% predictive value of RWCP on AF recurrence risk in different models. [file JCE-36-2885-s001.docx]

**Table S1 The distribution of RWCP scores for each group along with the results of one-way ANOVA**

| Groups | N | Mean±SD | Median (INR) | P-value |
| --- | --- | --- | --- | --- |
| A | 28 | -13.38 ± 6.43 | -14.93 (-18.26, -8.23) | 0.425 |
| B | 30 | -10.53 ± 7.56 | -7.95 (-16.81, -5.28) |  |
| C | 34 | -12.27 ± 7.06 | -12.46 (-17.09, -9.02) |  |
| D | 56 | -12.40 ± 6.01 | -13.23 (-17.25, -7.64) |  |

Abbreviations: N, Numbers; SD, standard deviation; Group A, neither side attained single-circle isolation; Group B, right side did not attain single-circle isolation while the left did; Group C, left side did not attain single-circle isolation while the right did; Group D, both sides attained single-circle isolation.

**Table S2 The further inter-group comparison results**

| Groups | Groups | MD (95% CI) | P-value |
| --- | --- | --- | --- |
| B | A | 2.85 (-1.70, 7.41) | 0.367 |
| C | A | 1.10 (-3.32, 5.53) | 0.916 |
| D | A | 0.98 (-3.03, 4.99) | 0.920 |
| C | B | -1.75 (-6.09, 2.60) | 0.723 |
| D | B | -1.87 (-5.79, 2.05) | 0.604 |
| D | C | -0.12 (-3.89, 3.65) | 1.000 |

Abbreviations: MD, mean deviation; Group A, neither side attained single-circle isolation; Group B, right side did not attain single-circle isolation while the left did; Group C, left side did not attain single-circle isolation while the right did; Group D, both sides attained single-circle isolation.

**Table S3 Included whether the left or right isolation achieved single-circle isolation in the multivariate Cox regression analysis**

| Variable | Fully adjusted model |
| --- | --- |
|  | (HR, 95%CI, P) |
| RWCP score (consistent) | 1.10 (1.04, 1.16) 0.001 |
| RWCP score (tertiles) |  |
| Low (-28.68 to -16.68) | 1 |
| Medium (-16.51 to -9.34) | 2.19 (0.77, 6.24) 0.143 |
| High (-9.02 to 5.79) | 3.26 (1.20, 8.90) 0.021 |
| RWCP score (median) |  |
| Low (-28.68 to -12.76) | 1 |
| High (-12.53 to 5.79) | 5.46 (2.174, 3.74) 0.001 |

Crude model: Fully adjusted model: In addition to age and sex, AF onset type and LAD were also adjusted.

Abbreviation: RWCP, real-world cardiologist performance; HR, hazard ratio; CI, confidence interval.


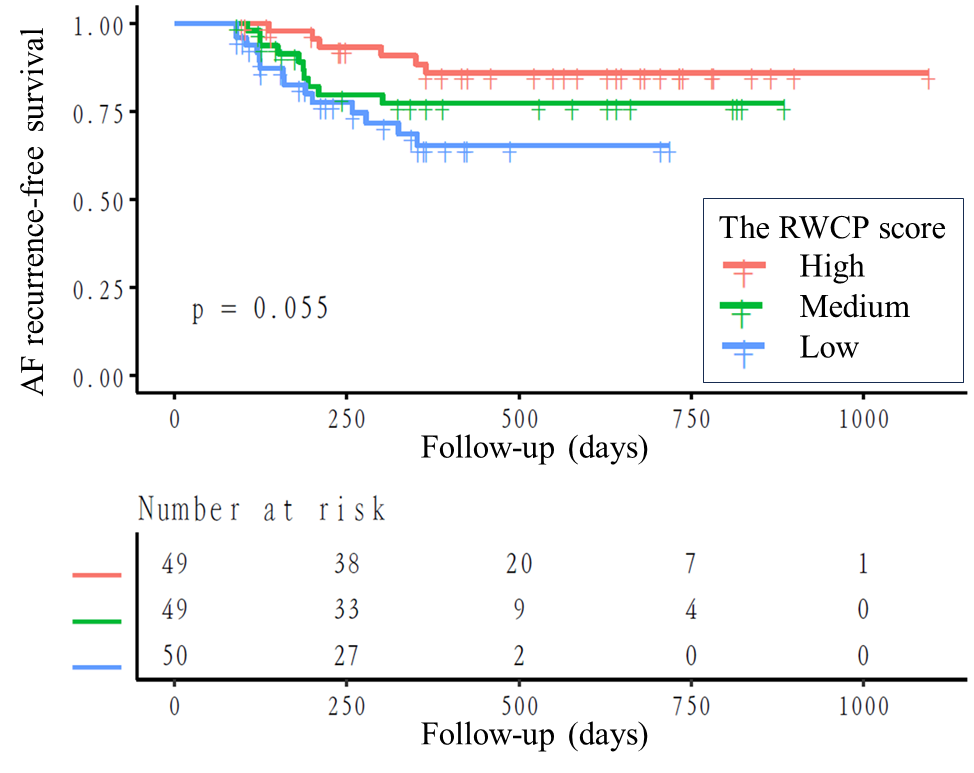
**Figure S1 Kaplan-Meier analysis (grouped by tertiles)**

Kaplan-Meier survival analysis demonstrated a clear trend in survival curves across the three groups (high, medium, and low RWCP score). However, the log-rank test for the three groups did not reach statistical significance.

|  | Non-recurrence | Recurrence | P-value |
| --- | --- | --- | --- |
|  | (n=100) | (n=22) |  |
| Gender (n,%) |  |  | 0.369 |
| Female | 44 (44.00%) | 12 (54.55%) |  |
| Male | 56 (56.00%) | 10 (45.45%) |  |
| Age (year) | 62.26 ± 5.87 | 60.50 ± 4.83 | 0.192 |
| BMI (Kg/m^2) | 25.22 ± 3.05 | 25.40 ± 5.01 | 0.831 |
| Smoking (n,%) | 29 (29.00%) | 5 (22.73%) | 0.552 |
| Drinking (n,%) | 29 (29.00%) | 11 (50.00%) | 0.057 |
| HBP (n,%) | 48 (48.00%) | 12 (54.55%) | 0.578 |
| DM (n,%) | 15 (15.00%) | 2 (9.09%) | 0.469 |
| CAD | 15 (15.00%) | 1 (4.55%) | 0.188 |
| Stroke/TIA (n,%) | 6 (6.00%) | 2 (9.09%) | 0.596 |
| AF duration (month) | 20.71 ± 34.17 | 26.77 ± 38.15 | 0.706 |
| TTE parameters |  |  |  |
| LAD (mm) | 39.03 ± 4.88 | 38.50 ± 5.74 | 0.656 |
| LVEDD (mm) | 46.03 ± 4.35 | 45.55 ± 4.68 | 0.642 |
| LVEF (%) | 65.79 ± 5.89 | 65.32 ± 5.39 | 0.731 |
| CAD (n,%) | 39.03 ± 4.88 | 38.50 ± 5.74 | 0.656 |
| Stroke/TIA (n,%) | 46.03 ± 4.35 | 45.55 ± 4.68 | 0.642 |
| AF duration (month) | 65.79 ± 5.89 | 65.32 ± 5.39 | 0.731 |
| Left ring |  |  |  |
| Discharge time proportion | 39.24 ± 14.27 | 39.10 ± 9.75 | 0.965 |
| C3-FOT proportion | 95.25 ± 10.90 | 95.56 ± 5.92 | 0.897 |
| SURPOINT/TOP-4 proportion | 87.84 ± 11.72 | 90.79 ± 7.04 | 0.260 |
| Fragmented points proportion | 9.15 ± 12.84 | 7.29 ± 7.98 | 0.768 |
| Right ring |  |  |  |
| Discharge time proportion | 40.19 ± 12.74 | 37.76 ± 10.83 | 0.408 |
| C3-FOT proportion | 95.53 ± 5.38 | 93.06 ± 7.97 | 0.078 |
| SURPOINT/TOP-4 proportion | 93.91 ± 8.21 | 95.53 ± 5.20 | 0.378 |
| Fragmented points proportion | 4.77 ± 9.79 | 4.03 ± 7.99 | 0.476 |
| Groups (single-circle isolation) |  |  | 0.296 |
| A | 23 (23.00%) | 3 (13.64%) |  |
| B | 19 (19.00%) | 7 (31.82%) |  |
| C | 18 (18.00%) | 6 (27.27%) |  |
| D | 40 (40.00%) | 6 (27.27%) |  |

**Table S4 Baseline information and ablation parameters of PAF patients**

Abbreviations: PAF, paroxysmal atrial fibrillation; BMI, body mass index; HBP, high blood pressure; DM, diabetes mellitus; CAD, coronary heart disease; TIA, transient ischemic attack; AF, atrial fibrillation; TTE, transthoracic echocardiography; LAD, left atrial diameter; LVEDD, left ventricular end-diastolic diameter; LVEF, left ventricular ejection fraction; Group A, neither side attained single-circle isolation; Group B, right side did not attain single-circle isolation while the left did; Group C, left side did not attain single-circle isolation while the right did; Group D, both sides attained single-circle isolation.

**Table S5 The tertiles and regression coefficients for ablation parameters in PAF patients**

| Ablation parameters | T1 (n, range) | T2 (n, range) | T3 (n, range) | RC*10 |
| --- | --- | --- | --- | --- |
| Left ring |  |  |  |  |
| Discharge time proportion | 41 (15.10-32.10) | 40 (32.70-43.40) | 41 (43.80-100.00) | 0.050 |
| C3-FOT proportion | 39 (0.00-95.50) | 33 (95.70-98.10) | 50 (100.00-100.00) | -1.350 |
| SURPOINT/TOP-4 proportion | 40 (14.50-87.00) | 40 (87.50-93.50) | 42 (93.90-100.00) | 4.833 |
| Fragmented points proportion | 61 (0.00-3.40) | 61 (4.40-85.50) |  | 5.523 |
| Right ring |  |  |  |  |
| Discharge time proportion | 41 (19.00-33.40) | 40 (33.70-43.40) | 41 (44.00-85.50) | -2.191 |
| C3-FOT proportion | 41 (69.40-93.90) | 36 (94.10-98.10) | 45 (100.00-100.00) | -3.204 |
| SURPOINT/TOP-4 proportion | 39 (35.40-93.50) | 42 (93.90-97.50) | 41 (97.60-100.00) | 0.373 |
| Fragmented points proportion | 78 (0.00-0.00) | 44 (2.10-74.40) |  | -4.538 |

Abbreviations: RC, regression coefficient.

**Table S6 The results of univariate cox regression analysis in PAF patients**

| Parameters | HR (95%CI) | P value |
| --- | --- | --- |
| Gender | 0.71 (0.31, 1.64) | 0.418 |
| Age | 0.96 (0.89, 1.03) | 0.254 |
| BMI | 1.02 (0.90, 1.15) | 0.794 |
| Smoking | 0.68 (0.25, 1.83) | 0.442 |
| Drinking | 1.86 (0.81, 4.29) | 0.146 |
| AF duration | 1.00 (1.00, 1.01) | 0.364 |
| HBP | 1.39 (0.60, 3.21) | 0.445 |
| DM | 0.93 (0.22, 3.98) | 0.920 |
| CAD | 0.36 (0.05, 2.65) | 0.314 |
| Stroke/TIA | 3.12 (0.72, 13.54) | 0.128 |
| LAD | 0.97 (0.89, 1.06) | 0.497 |
| LVEDD | 0.97 (0.88, 1.07) | 0.567 |
| LVEF | 1.01 (0.94, 1.09) | 0.809 |
| Groups (single-circle isolation) |  |  |
| A | 1 |  |
| B | 2.56 (0.66, 9.91) | 0.173 |
| C | 2.88 (0.72, 11.55) | 0.135 |
| D | 1.27 (0.32, 5.08) | 0.736 |

Abbreviations: BMI, body mass index; AF, atrial fibrillation; HBP, high blood pressure; DM, diabetes mellitus; CAD, coronary heart disease; TIA, transient ischemic attack; LAD, left atrial diameter; LVEDD, left ventricular end-diastolic diameter; LVEF, left ventricular ejection fraction; Group A, neither side attained single-circle isolation; Group B, right side did not attain single-circle isolation while the left did; Group C, left side did not attain single-circle isolation while the right did; Group D, both sides attained single-circle isolation.

**Table S7 PAF patients’ predictive value of RWCP on AF recurrence risk**

| Variable | Crude model | Minimally adjusted model | Fully adjusted model |
| --- | --- | --- | --- |
|  | (HR, 95%CI, P) | (HR, 95%CI, P) | (HR, 95%CI, P) |
| RWCP score (consistent) | 1.11(1.02, 1.20) 0.017 | 1.12 (1.03, 1.22) 0.011 | 1.12 (1.03, 1.22) 0.012 |
| RWCP score (tertiles) |  |  |  |
| Low (-15.22 to -2.65) | 1 | 1 | 1 |
| Medium (-2.56 to 1.92) | 1.25 (0.36, 4.31) 0.727 | 1.46 (0.41, 5.20) 0.563 | 1.50 (0.42, 5.43) 0.534 |
| High (2.13 to 14.31) | 3.43 (1.20, 9.77) 0.021 | 4.03 (1.35, 11.99) 0.012 | 4.08 (1.36, 12.24) 0.012 |
| RWCP score (median) |  |  |  |
| Low (-15.22 to -0.08) | 1 | 1 | 1 |
| High (-0.03 to 14.31) | 1.92 (0.82, 4.49) 0.135 | 2.14 (0.89, 5.16) 0.091 | 2.14 (0.88, 5.19) 0.092 |

Crude model: no covariate adjusted. Minimally adjusted model: only age and sex adjusted. Fully adjusted model: In addition to age and sex, AF onset type and LAD were also adjusted.)

Abbreviation: RWCP, real-world cardiologist performance; HR, hazard ratio; CI, confidence interval.


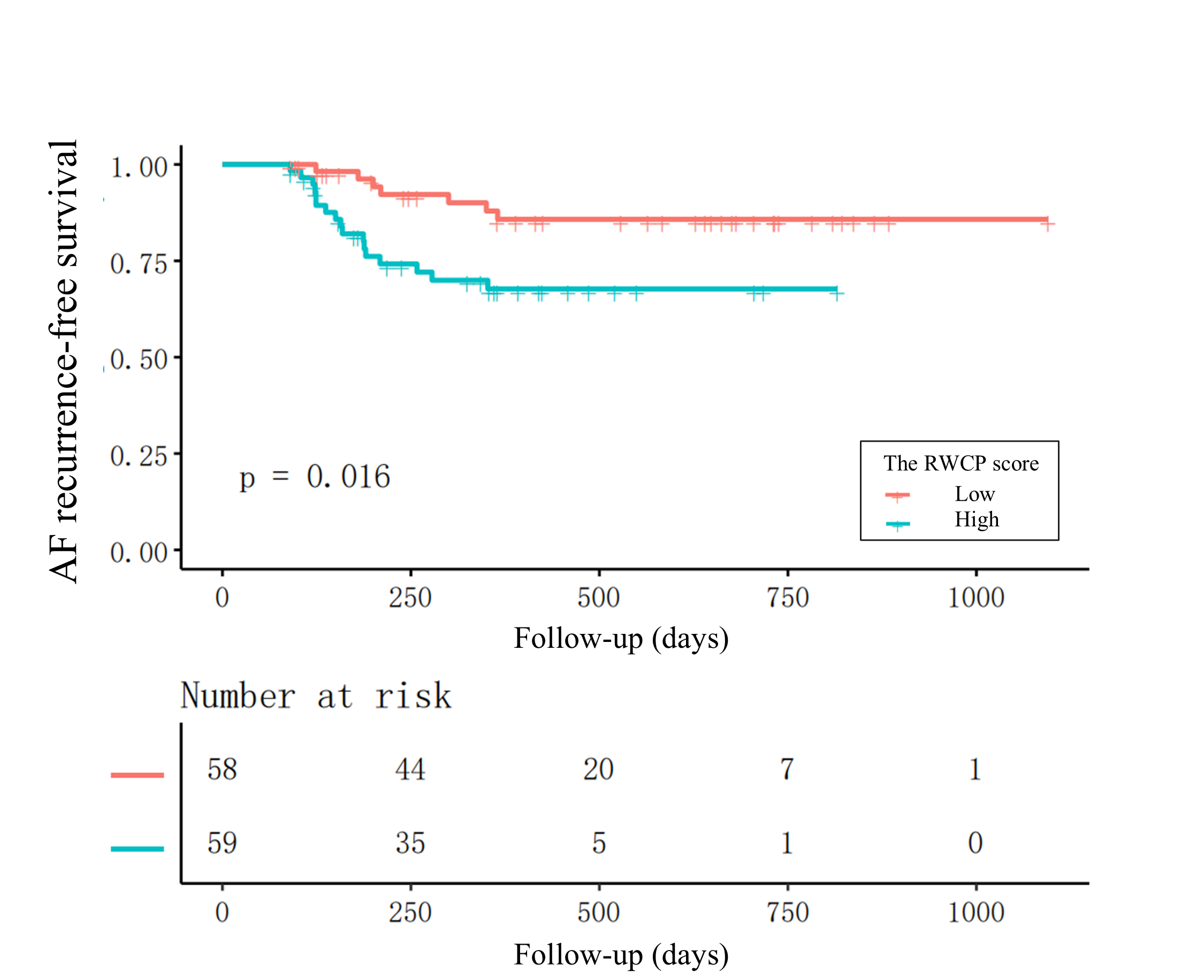


**Figure S2 The Kaplan-Meier analysis of two groups in PAF**

Kaplan-Meier survival analysis demonstrated a clear trend in survival curves across the two groups (low and high RWCP score) in PAF. The log-rank test for the two groups reach statistical significance (P_log-rank_<0.05).


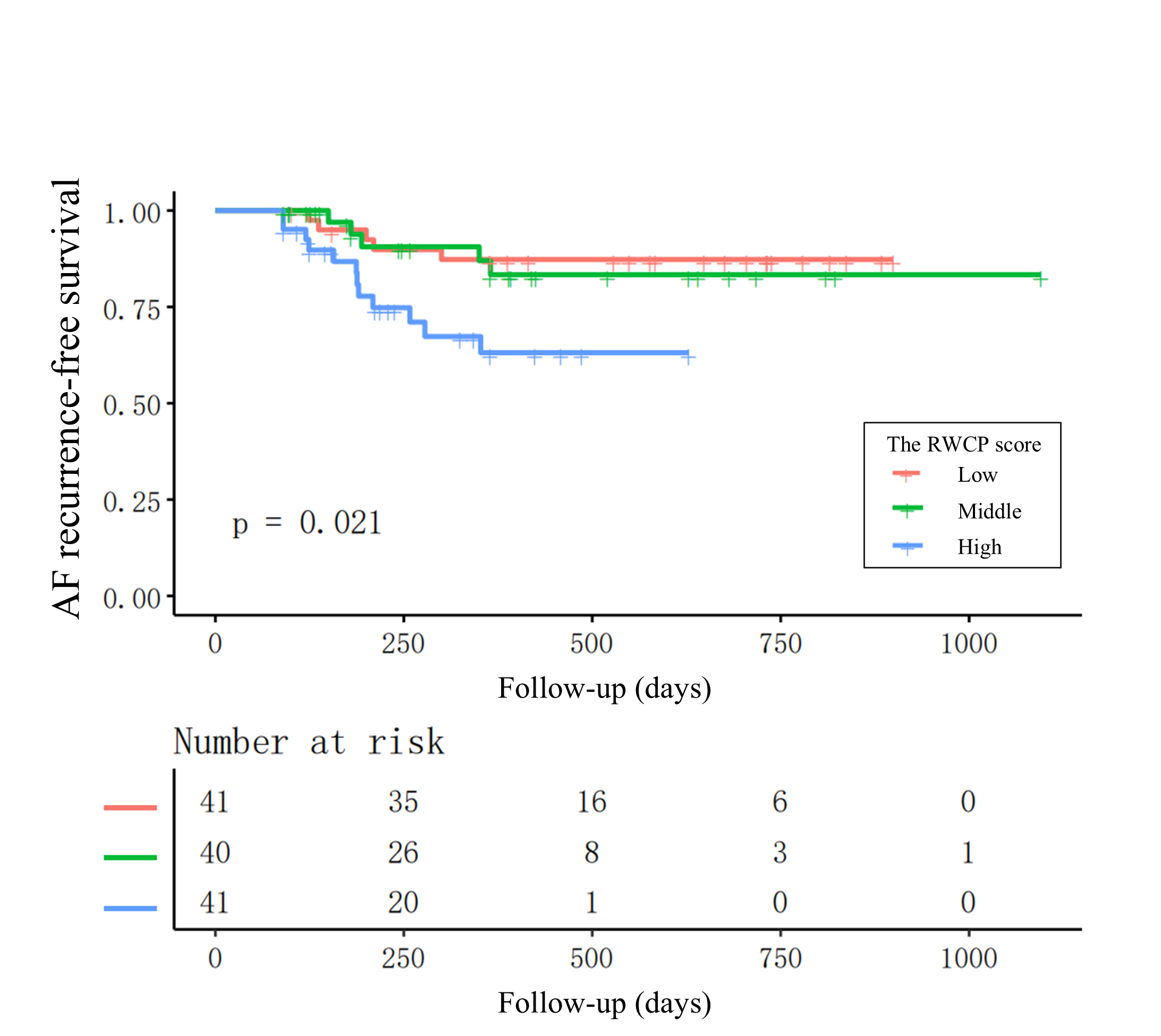


**Figure S3 The Kaplan-Meier analysis of three groups in PAF**

Kaplan-Meier survival analysis demonstrated a clear trend in survival curves across the three groups (low, medium and high RWCP score) in PAF. The log-rank test for the three groups reach statistical significance (p_log-rank_<0.05).

**Table S8 Baseline information and ablation parameters among patients with low voltage areas less than 20%**

|  | Non-recurrence (n=93) | Recurrence (n=24) | P-value |
| --- | --- | --- | --- |
| Gender (n,%) |  |  | 0.431 |
| Female | 42 (45.16%) | 13 (54.17%) |  |
| Male | 51 (54.84%) | 11 (45.83%) |  |
| Age (year) | 61.90 ± 5.38 | 60.63 ± 4.71 | 0.289 |
| BMI (Kg/m^2) | 25.25 ± 3.19 | 25.48 ± 4.81 | 0.779 |
| Smoking (n,%) | 26 (27.96%) | 5 (20.83%) | 0.481 |
| Drinking (n,%) | 30 (32.26%) | 9 (37.50%) | 0.627 |
| HBP (n,%) | 43 (46.24%) | 13 (54.17%) | 0.488 |
| DM (n,%) | 11 (11.83%) | 3 (12.50%) | 0.928 |
| CAD (n,%) | 12 (12.90%) | 1 (4.17%) | 0.225 |
| Stroke/TIA (n,%) | 6 (6.45%) | 1 (4.17%) | 0.674 |
| AF onset type (n,%) |  |  | 0.632 |
| PAF | 81 (87.10%) | 20 (83.33%) |  |
| PsAF | 12 (12.90%) | 4 (16.67%) |  |
| AF duration (month) | 20.14 ± 32.73 | 25.88 ± 36.98 | 0.786 |
| TTE parameters |  |  |  |
| LAD (mm) | 39.28 ± 4.84 | 38.13 ± 5.14 | 0.306 |
| LVEDD (mm) | 46.09 ± 4.49 | 45.42 ± 4.40 | 0.514 |
| LVEF (%) | 65.74 ± 5.92 | 66.38 ± 5.67 | 0.638 |
| Left ring |  |  |  |
| Discharge time proportion | 37.98 ± 14.14 | 38.56 ± 9.82 | 0.850 |
| C3-FOT proportion | 95.57 ± 11.14 | 96.49 ± 5.08 | 0.694 |
| SURPOINT/TOP-4 proportion | 87.45 ± 11.93 | 90.43 ± 7.23 | 0.244 |
| Fragmented points proportion | 9.63 ± 13.55 | 7.75 ± 8.30 | 0.844 |
| Right ring |  |  |  |
| Discharge time proportion | 39.60 ± 11.90 | 37.48 ± 10.36 | 0.425 |
| C3-FOT proportion | 95.67 ± 5.35 | 93.89 ± 5.68 | 0.155 |
| SURPOINT/TOP-4 proportion | 93.73 ± 8.25 | 94.93 ± 5.46 | 0.501 |
| Fragmented points proportion | 5.25 ± 10.19 | 3.47 ± 7.64 | 0.235 |
| Groups (single-circle isolation) |  |  | 0.067 |
| A | 20 (21.51%) | 3 (12.50%) |  |
| B | 18 (19.36%) | 9 (37.50%) |  |
| C | 16 (17.20%) | 7 (29.17%) |  |
| D | 39 (41.94%) | 5 (20.83%) |  |

Abbreviations: BMI, body mass index; HBP, high blood pressure; DM, diabetes mellitus; CAD, coronary heart disease; TIA, transient ischemic attack; AF, atrial fibrillation; TTE, transthoracic echocardiography; LAD, left atrial diameter; LVEDD, left ventricular end-diastolic diameter; LVEF, left ventricular ejection fraction; Group A, neither side attained single-circle isolation; Group B, right side did not attain single-circle isolation while the left did; Group C, left side did not attain single-circle isolation while the right did; Group D, both sides attained single-circle isolation.

**Table S9 The tertiles and regression coefficients for ablation parameters among**

**patients with low voltage areas less than 20%**

| Ablation parameters | T1 (n, range) | T2 (n, range) | T3 (n, range) | RC*10 |
| --- | --- | --- | --- | --- |
| Left ring |  |  |  |  |
| Discharge time proportion | 41 (15.10-32.10) | 40 (32.70-43.40) | 41 (43.80-100.00) | 2.029 |
| C3-FOT proportion | 39 (0.00-95.50) | 33 (95.70-98.10) | 50 (100.00-100.00) | 0.854 |
| SURPOINT/TOP-4 proportion | 40 (14.50-87.00) | 40 (87.50-93.50) | 42 (93.90-100.00) | 4.654 |
| Fragmented points proportion | 61 (0.00-3.40) | 61 (4.40-85.50) |  | 6.525 |
| Right ring |  |  |  |  |
| Discharge time proportion | 41 (19.00-33.40) | 40 (33.70-43.40) | 41 (44.00-85.50) | -2.808 |
| C3-FOT proportion | 41 (69.40-93.90) | 36 (94.10-98.10) | 45 (100.00-100.00) | -4.836 |
| SURPOINT/TOP-4 proportion | 39 (35.40-93.50) | 42 (93.90-97.50) | 41 (97.60-100.00) | -0.586 |
| Fragmented points proportion | 78 (0.00-0.00) | 44 (2.10-74.40) |  | -6.300 |

Abbreviations: RC, regression coefficient.

**Table S10 The results of univariate cox regression analysis among patients with low voltage areas less than 20%**

| Parameters | HR (95%CI) | P value |
| --- | --- | --- |
| Gender | 0.82 (0.37, 1.84) | 0.635 |
| Age | 0.96 (0.89, 1.04) | 0.303 |
| BMI | 1.02 (0.92, 1.14) | 0.702 |
| Smoking | 0.67 (0.25, 1.79) | 0.422 |
| Drinking | 1.03 (0.45, 2.35) | 0.950 |
| PsAF | 1.31 (0.45, 3.84) | 0.621 |
| AF duration | 1.00 (1.00, 1.01) | 0.415 |
| HBP | 1.40 (0.63, 3.13) | 0.413 |
| DM | 1.80 (0.53, 6.07) | 0.347 |
| CAD | 0.35 (0.05, 2.57) | 0.300 |
| Stroke/TIA | 1.26 (0.17, 9.37) | 0.825 |
| LAD | 0.96 (0.88, 1.04) | 0.298 |
| LVEDD | 0.97 (0.88, 1.06) | 0.476 |
| LVEF | 1.04 (0.96, 1.12) | 0.329 |
| Groups (single-circle isolation) |  |  |
| A | 1 |  |
| B | 2.95 (0.80, 10.90) | 0.105 |
| C | 3.07 (0.79, 11.87) | 0.105 |
| D | 0.96 (0.23, 4.03) | 0.957 |

Abbreviations: BMI, body mass index; AF, atrial fibrillation; HBP, high blood pressure; DM, diabetes mellitus; CAD, coronary heart disease; TIA, transient ischemic attack; LAD, left atrial diameter; LVEDD, left ventricular end-diastolic diameter; LVEF, left ventricular ejection fraction; Group A, neither side attained single-circle isolation; Group B, right side did not attain single-circle isolation while the left did; Group C, left side did not attain single-circle isolation while the right did; Group D, both sides attained single-circle isolation.

**Table S11 Among patients’with low voltage areas less than 20% predictive value of RWCP on AF recurrence risk in different models**

| Variable | Crude model | Minimally adjusted model | Fully adjusted model |
| --- | --- | --- | --- |
|  | (HR, 95%CI, P) | (HR, 95%CI, P) | (HR, 95%CI, P) |
| RWCP score (consistent) | 1.10 (1.04, 1.17) 0.001 | 1.11 (1.05, 1.18) 0.001 | 1.12 (1.05, 1.19) 0.001 |
| RWCP score (tertiles) |  |  |  |
| Low (-17.317 to -3.210) | 1 | 1 | 1 |
| Medium (-2.862 to 2.541) | 3.74 (1.03, 13.61) 0.045 | 4.15 (1.12, 15.35) 0.033 | 4.56 (1.21, 17.20) 0.025 |
| High (2.591 to 20.644) | 4.90 (1.36, 17.61) 0.015 | 5.31 (1.47, 19.23) 0.011 | 5.95 (1.61, 22.07) 0.008 |
| RWCP score (median) |  |  |  |
| Low (-17.317 to -0.232) | 1 | 1 | 1 |
| High (-0.164 to 20.644) | 2.82 (1.17, 6.80) 0.021 | 2.88 (1.19, 6.96) 0.019 | 3.17 (1.29, 7.81) 0.012 |

Crude model: no covariate adjusted. Minimally adjusted model: only age and sex adjusted. Fully adjusted model: In addition to age and sex, AF onset type and LAD were also adjusted.)

Abbreviation: RWCP, real-world cardiologist performance; HR, hazard ratio; CI, confidence interval.


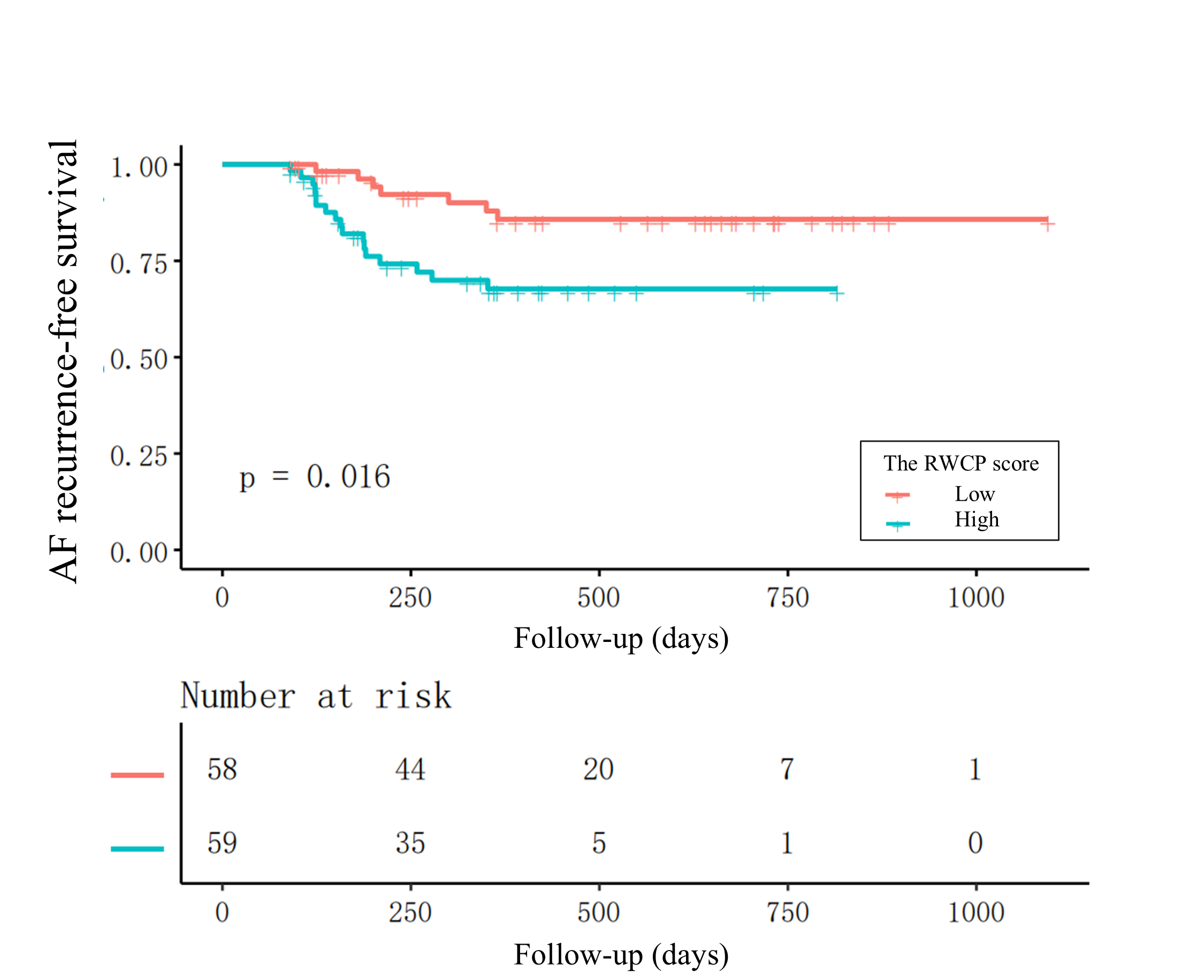


**Figure S4 The Kaplan-Meier analysis of two groups among patients with low voltage areas less than 20%**

Kaplan-Meier survival analysis of two groups among patients with low voltage areas less than 20% demonstrated a clear trend in survival curves across the two groups (low and high RWCP score). The log-rank test for the two groups reach statistical significance (p_log-rank_<0.05).


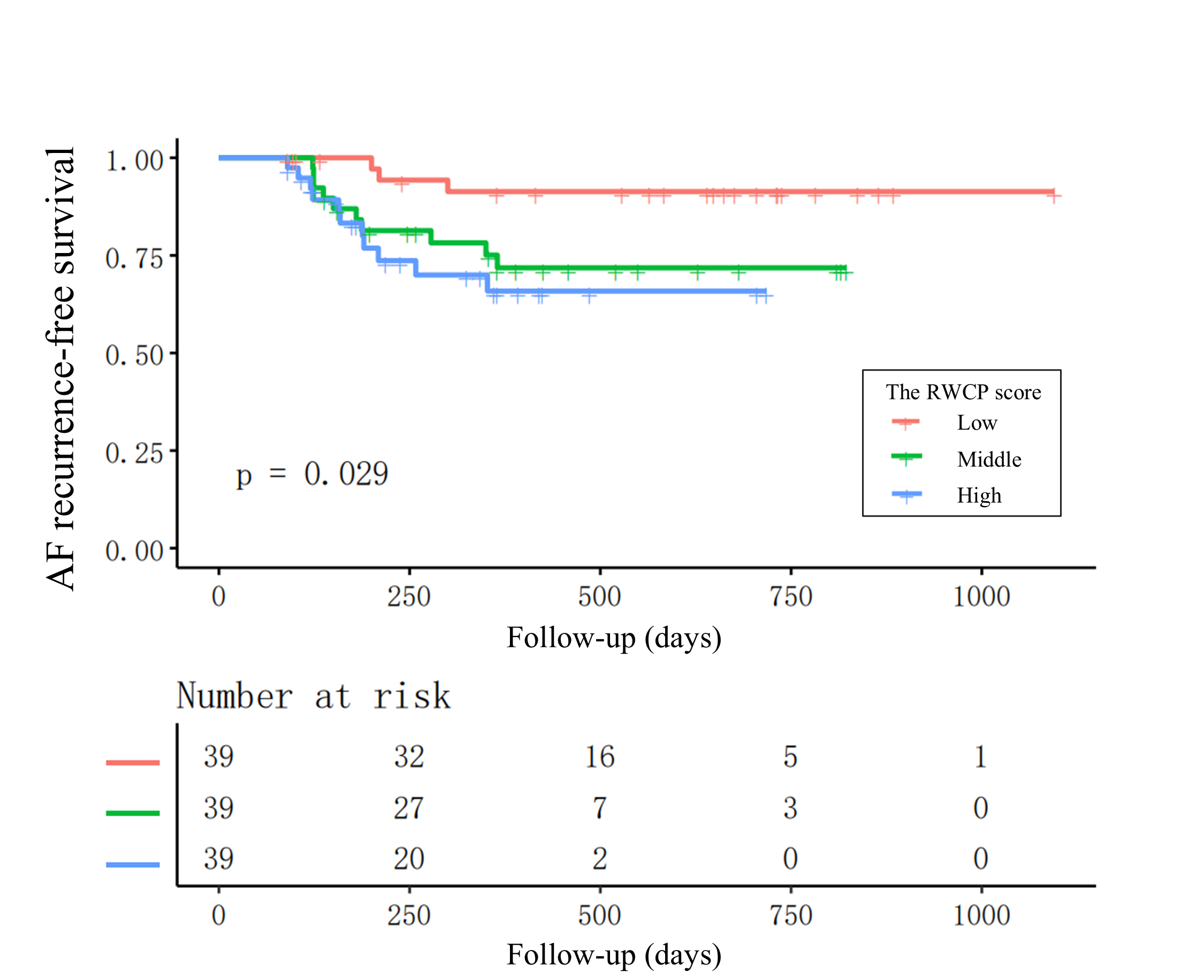


**Figure S5 The Kaplan-Meier analysis of three groups among patients with low voltage areas less than 20%.**

Kaplan-Meier survival analysis of three groups among patients with low voltage areas less than 20% demonstrated a clear trend in survival curves across the three groups (low, medium and high RWCP score). The log-rank test for the three groups reach statistical significance (p_log-rank_<0.05).
