# Supplementary figures and images for: The Predictive Value of Real‐World Cardiologist Performance (RWCP) Score in Atrial Fibrillation Recurrence Risk After Radiofrequency Ablation
Source: J Cardiovasc Electrophysiol. 2025 Aug 19;36(11):2885–93. doi: 10.1111/jce.70067 (PMC12614126; doi:10.1111/jce.70067)

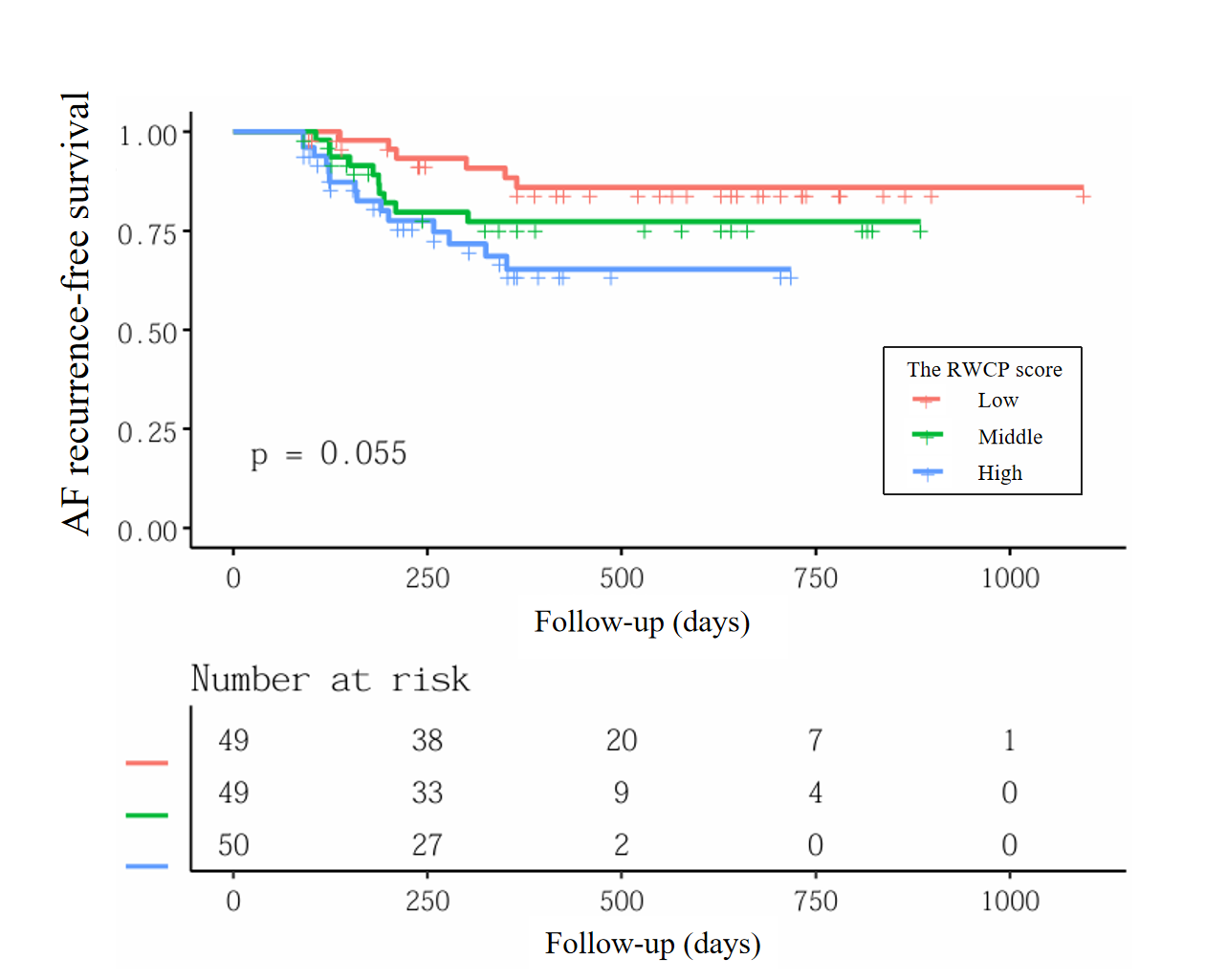

Supplement: Supplementary file 2 — Figure S1: Kaplan‐Meier analysis (grouped by tertiles). [file JCE-36-2885-s005.tif]

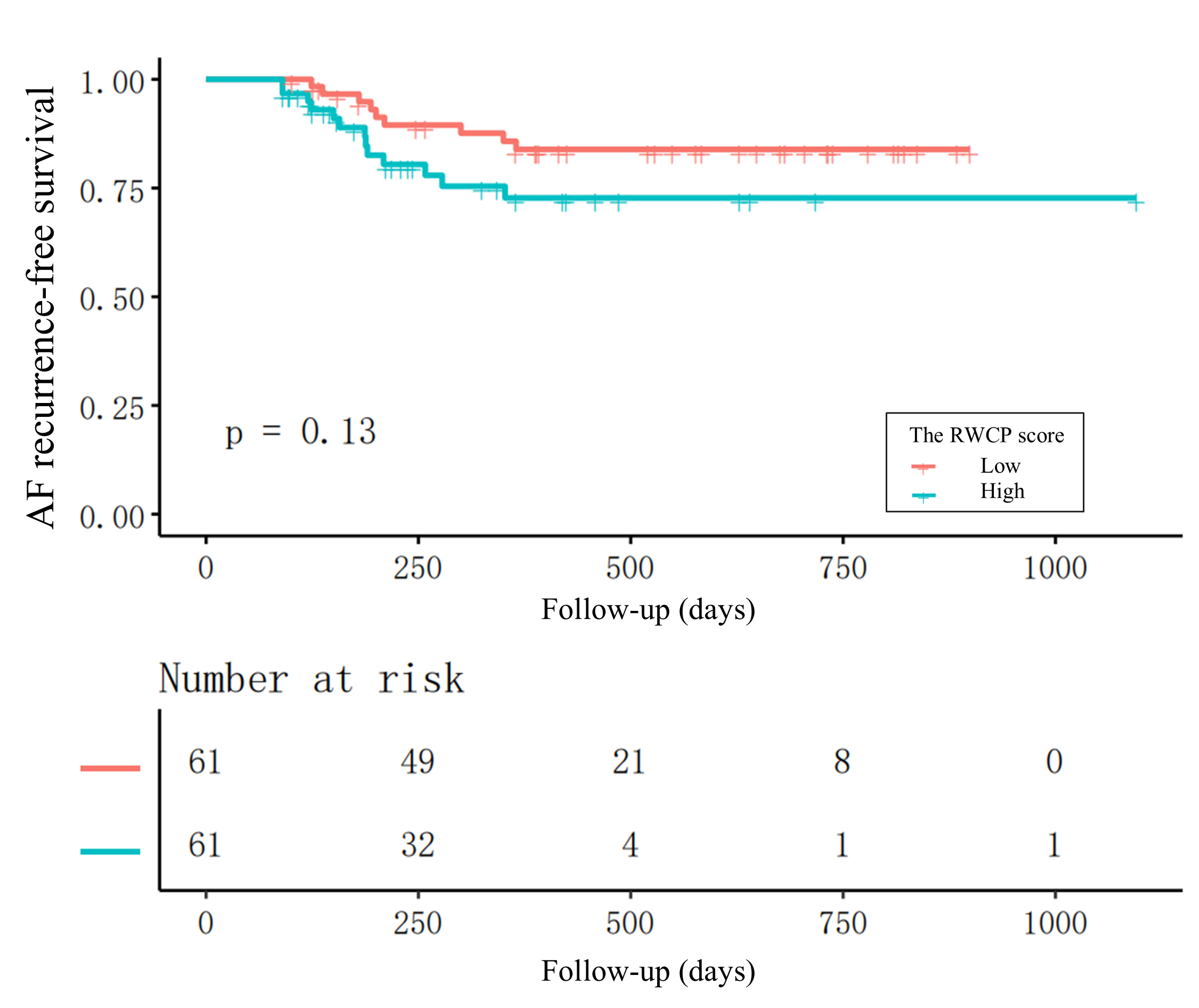

Supplement: Supplementary file 3 — Figure S2: The Kaplan‐Meier analysis of two groups in PAF. [file JCE-36-2885-s006.tif]

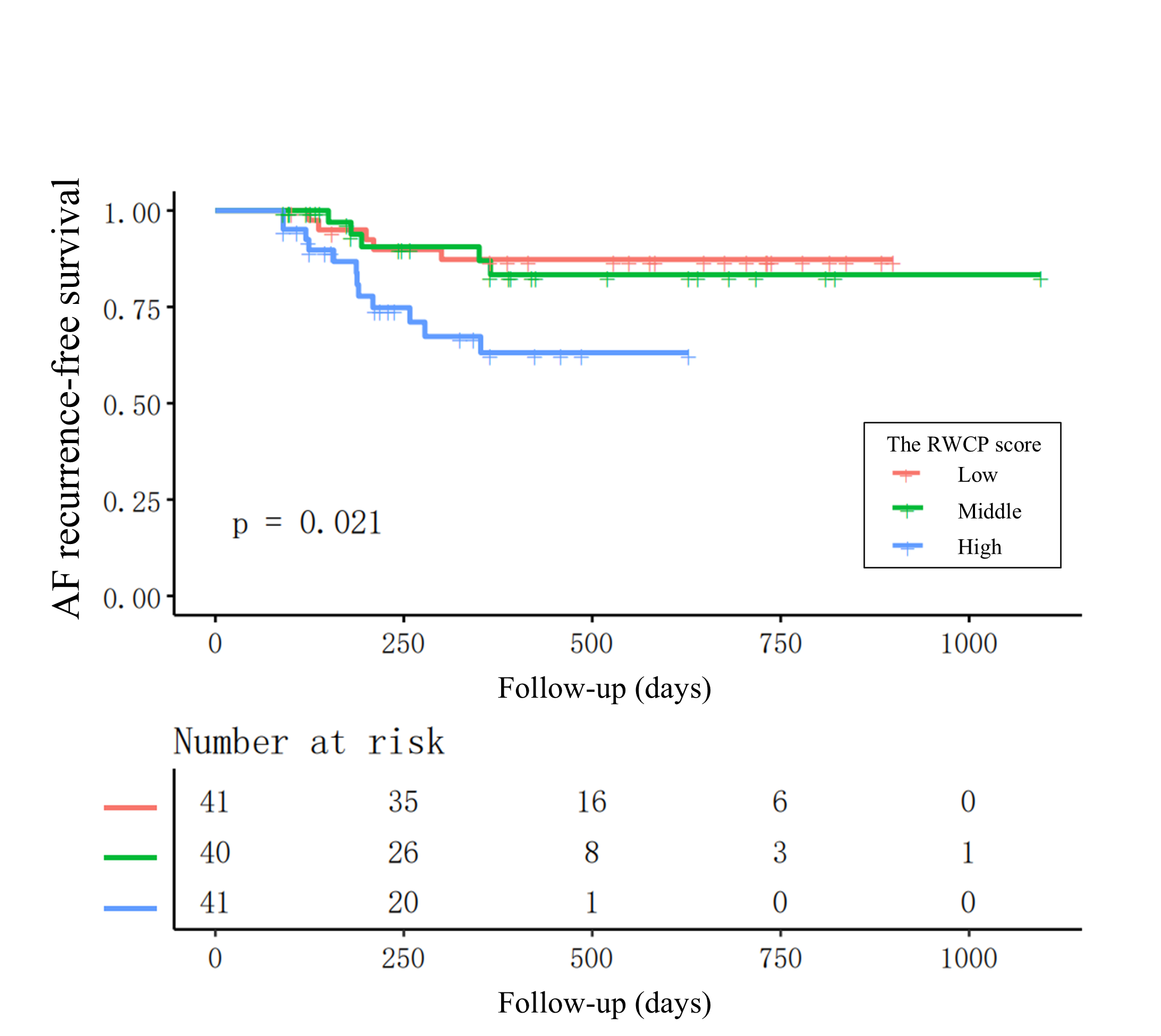

Supplement: Supplementary file 4 — Figure S3: The Kaplan‐Meier analysis of three groups in PAF. [file JCE-36-2885-s003.tif]

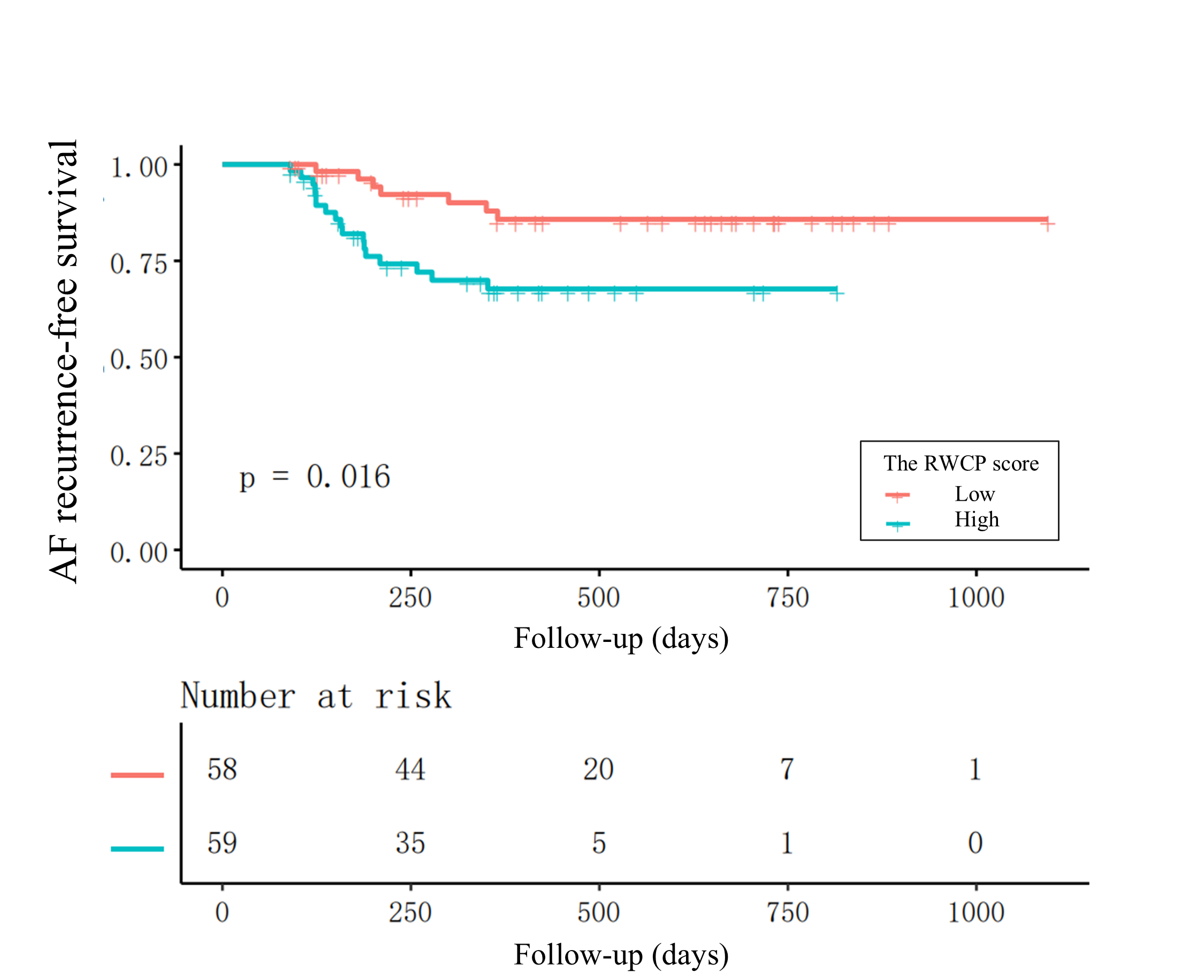

Supplement: Supplementary file 5 — Figure S4: The Kaplan‐Meier analysis of two groups among patients with low voltage areas less than 20%. [file JCE-36-2885-s002.tif]

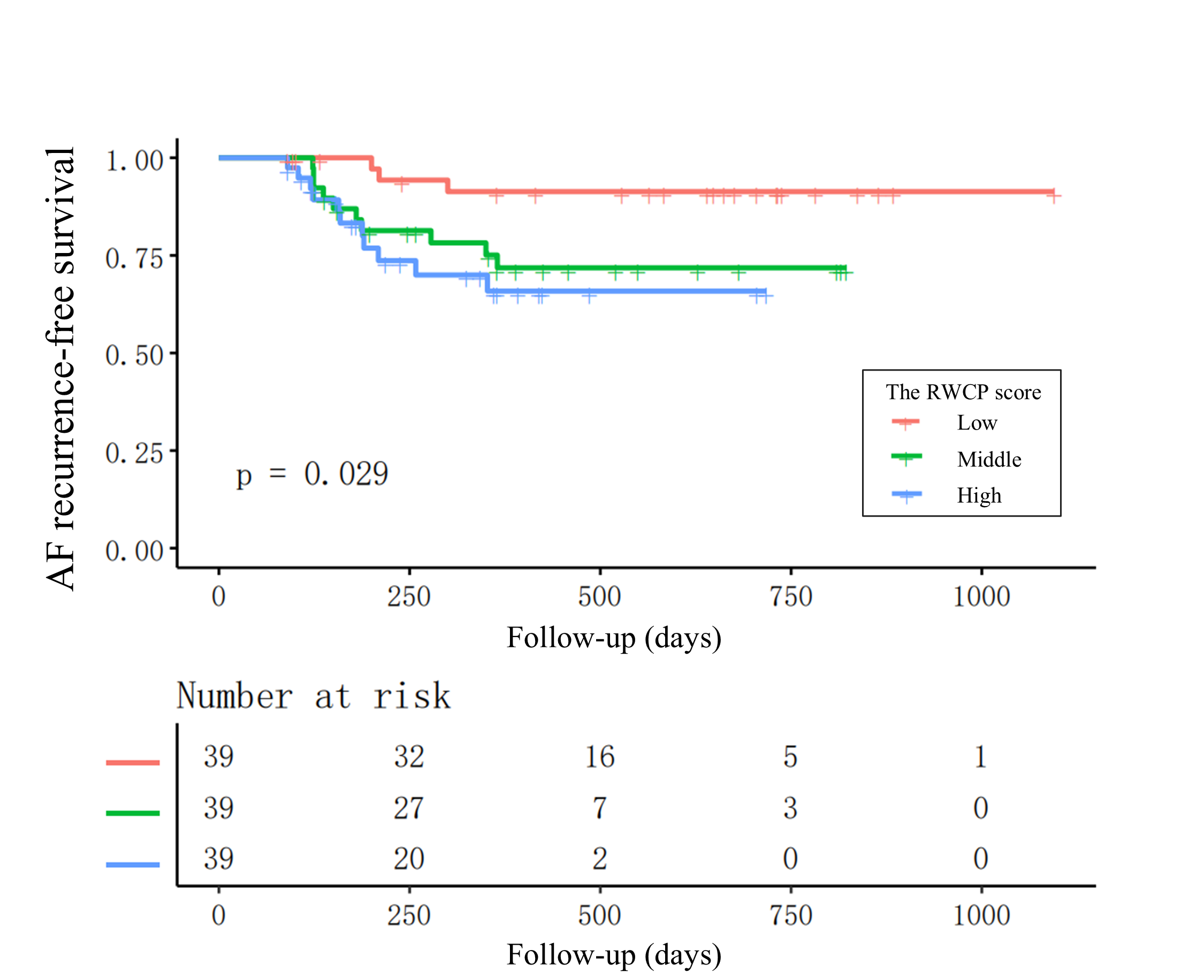

Supplement: Supplementary file 6 — Figure S5: The Kaplan‐Meier analysis of three groups among patients with low voltage areas less than 20%. [file JCE-36-2885-s004.tif]
